# A pre-vaccine exploratory survey of SARS-CoV-2 humoral immunity among Egyptian general population

**DOI:** 10.1186/s41182-022-00448-x

**Published:** 2022-08-10

**Authors:** Engy Mohamed El-Ghitany, Shehata Farag, Azza Galal Farghaly, Mona H. Hashish, Mahmoud A. Hassaan, Eman A. Omran

**Affiliations:** 1 Department of Tropical Health and Parasitology, High Institute of Public Health, Alexandria University, 165 El-Horreya Avenue-El-Ibrahimia, Alexandria, Egypt; 2 Department of Biostatistics, High Institute of Public Health, Alexandria University, Alexandria, Egypt; 3 Family and Community Medicine Department, Faculty of Medicine, King Khalid University, Abha, Saudi Arabia; 4 Department of Microbiology, High Institute of Public Health, Alexandria University, Alexandria, Egypt; 5 Institute of Graduate Studies and Research, Alexandria University, Alexandria, Egypt

**Keywords:** SARS-CoV-2, Seroprevalence, Anti-spike, Immune-response

## Abstract

**Background:**

Population-based studies on COVID-19 have important implications for modeling the pandemic and determining vaccination policies. Limited data are available from such surveys in Egypt.

**Methods:**

This cross-sectional was conducted throughout the period between January and June 2021, which coincided with the second and third waves of the COVID-19 pandemic in Egypt. At that time, vaccines against COVID-19 were not available to the general population. The study was carried out in eight Egyptian governorates and included 2360 participants, who were recruited through a multistage stratified cluster sample technique, based on gender, age, and district followed by a random sample within each district. Socio-demographic data were recorded and serum samples were collected and tested for SARS-Co-V2 spike (S) antibodies.

**Results:**

The overall adjusted prevalence of anti-S was 46.3% (95% CI 44.2–48.3%), with significant differences between governorates. Factors associated with anti-S seropositivity were: being female (*p* = 0.001), living in a rural area (*p* = 0.008), and reporting a history of COVID-19 infection (*p* = 0.001). Higher medians of anti-S titers were significantly associated with: extremes of age (*p* < 0.001), living in urban areas, having primary education (*p* = 0.009), and reporting a history of COVID-19 infection, especially if based on chest CT or PCR (*p* < 0.001).

**Conclusions:**

High seroprevalence rates indicate increased COVID-19 infection and immune response among a considerable percentage of the community. Age, gender, residence, educational level, and previous PCR-confirmed COVID-19 infections were all determinants of the immune response.

**Supplementary Information:**

The online version contains supplementary material available at 10.1186/s41182-022-00448-x.

## Background

The severe acute respiratory syndrome coronavirus-2 (SARS-CoV-2) has become the most urgent public health problem worldwide, causing the coronavirus disease 2019 (COVID-19) pandemic [1]. Globally, as of 29 July 2022, there have been 572,239,451 confirmed cases of COVID-19, including 6,390,401 deaths, reported to the World Health Organization (WHO) [2]. According to the WHO reports, Egypt recorded a total of 513,881 COVID-19 cases and 24,690 mortalities until May 22, 2022 [2]. In Egypt, the first COVID-19 wave began in March 2020, the second began in November 2020, and the third wave began by the end of March 2021 [3–5]. According to the WHO records, as of May 22, 2022, Egypt recorded no cases of COVID-19 within the last 24 h [2], indicating a currently declining pandemic, in accordance with the situation globally.

Following infection by SARS-CoV-2, antibodies are often positive, even among those showing no or minor symptoms. Serological assays for SARS-CoV-2 include direct and indirect assays, with variable sensitivity and specificity. Direct immunoassays, which include two antibody–antigen binding reactions, are correlated with the antibody affinity, while indirect immunoassays are better suited for quantifying anti-viral antibody levels [6]. An important serological marker for COVID-19 infection is the anti-spike (anti-S) antibodies, of which a subset is often able to neutralize the virus following its entry [7]. Furthermore, anti-S is considered an important serological domain in judging vaccine efficacy [8]. Anti-S is estimated to have a half-life of around 184 days [9].

Generally, mapping, recognizing, and analyzing the spatial pattern of seroprevalence can assist in delineating areas with high prevalence rates. Moreover, this can provide insight into the underlying factors that control these patterns. The current study aimed to explore the seroprevalence of SARS-CoV-2 spike antibodies as a crucial element of humoral immunity and identify the geographical distribution and socio-demographic determinants of SARS-CoV-2 infection. To the best of our knowledge, no such large-scale seroprevalence study has been carried out in various Egyptian governorates. This study was carried out prior to the availability of the COVID-19 vaccine, and thus antibody status reflected a previous infection rather than a vaccine response. Our study might help identify governorates with high infection rates and socio-demographic determinants of infection, thus helping to improve decision-making related to vaccine allocation and the application of preventive measures.

## Methods

This cross-sectional study was conducted throughout the period between January and June 2021. This period coincided with the second and third waves of the COVID-19 pandemic in Egypt. We aimed to identify the prevalence of COVID-19 seropositivity in several Egyptian governorates. All ages were included regardless of their history of previous COVID-19 infection and there were no exclusion criteria. None of the participants were vaccinated against SARS-CoV-2 at the study time, as the vaccine was not available to the public and was reserved primarily for healthcare workers, mainly those working in COVID-19 isolation hospitals.

### Study setting

Eight Egyptian governorates were included, six of them were from Lower Egypt, and two were from Upper Egypt (Giza and Faiyum).

### Sample size

Convenient sampling was adopted for participant allocation. The assumed sample size was large enough for an exploratory survey and suitable for the time factor needed to complete the survey. According to the basic tables for sample size estimation, the sample size was roughly estimated based on the population size and the acceptable margin of error. A total sample size of 1300 participants was required to estimate the average prevalence of SARS-CoV-2 antibodies of 6.9%, with a precision of 2% at a 95% confidence level and a design effect of 2. The sample size was calculated using Epi-Info 7 software using referenced parameters after an intensive literature review.

### Sampling technique

The study was conducted using a multistage stratified cluster sample technique. Stratification was done based on gender and age to include both genders and all age groups. The most affected districts within each governorate were included in the survey in the first stage. In the second stage, a random sample was included within each district based on the WHO method for surveying [10]. Within each district, four areas were chosen based on well-known landmarks of each area (hypermarkets, mosques, churches, or well-known buildings). In each selected district area, landmarks were chosen to invite all populations to participate in the survey. People were invited to participate in our survey through media announcement and distribution of flyers in the area by our team, explaining the aim and outcome of our survey. All persons who agreed to participate were included consecutively until the required sample size was achieved.

### Data collection methods and tools

A structured interview questionnaire sheet was designed and filled in for each participant, including governorate, residence (urban/rural/slum), age, sex, education, marital status, and history of COVID-19 diagnosis. For the purpose of our study, the presence of the following criteria (one or more of the parameters in **bold**) was considered as a positive history of COVID-19 infection:: **clinical** diagnosis by the treating physician based on symptoms (fever, cough, loss of taste and smell, myalgia, diarrhea), chest computed tomography (**CT**), **laboratory tests** (including elevated d-dimer, ferritin, erythrocyte sedimentation rate, leucopenia, lymphopenia or lymphocytosis), **rapid antigen test** and polymerase chain reaction (**PCR**) for SARS-CoV-2. Results of PCR included those that have been performed in governmental as well as private laboratories.

After obtaining written informed consent, a 3-ml venous blood sample was collected from each participant for anti-S testing. All 2360 samples were tested for anti-S. Serum samples were separated by centrifugation at 3000 rpm, and serum was stored frozen at – 20 °C until further processing.

The anti-SARS-CoV-2 Quantivac enzyme-linked immunosorbent assay (ELISA) (EuroImmun, Lübeck, Germany) was used for the quantitative detection of immunoglobulin class IgG against the S1 domain of the viral spike protein (including the receptor-binding domain; RBD). According to the manufacturer’s instructions, the results should be interpreted according to their relative unit (RU) results as follows: < 8 RU/ml were negative, while titers ≥ 8–< 11 RU/ml were borderline and those ≥ 11 RU/ml were considered positive. According to the manufacturer’s instructions, the sensitivity of this test is 93.2% after 21 days of symptom-onset and with a specificity of 99.8%.

For the convenience of statistical calculation of the median values of antibody levels, any values exceeding the value of the highest calibrator in the anti-S test (> 120 RU/ml) were considered 120 RU/ml.

### Data analysis

After the data were extracted, they were revised, coded, and fed to statistical software IBM SPSS version 22 (SPSS, Inc., Chicago, IL). All statistical analysis was done using two-tailed tests. Any *p*-value less than 0.05 was considered statistically significant. The frequency and percent distribution of descriptive analysis was done for all variables, including all eligible population socio-demographic data, screening results, and immunity status. Adjusted seropositive prevalence was calculated in addition to the crude prevalence to account for screening test sensitivity and specificity as the test validity measures are less than 100%, with some probability of false positive and false negative results [11].

Data acquired on the frequency of SARS-CoV-2 anti-S in the studied governorates were mapped using ArcGIS (ver. 10.8). Accordingly, thematic maps were produced representing the spatial pattern of SARS-CoV-2 anti-S rates and their relative distribution by gender and age groups in different governorates.

## Results

A total of 2360 participants were included from eight governorates, with significant differences in the characteristics of their residents regarding age, gender, educational level, marital status, and residence (Additional file 1: Table S1). The largest contingent of participants was from Alexandria (*n* = 715, 30.3%), followed by Monufia Governorate (*n* = 637, 27.0%). Females constituted 53.6% (*n* = 1264) of participants. One-third of the participants (*n* = 770, 32.6%) were from the age group 40–59 years, 19.4% were below 15 years (*n* = 457), and 12.1% (*n* = 286) were above 60 years of age. Urban residents constituted 51.2% (*n* = 1209) of the participants, while 37.1% (*n* = 875) were from rural areas and the rest from slums. Regarding educational level, 27.5% of participants (*n* = 648) received their secondary education, 26.5% (*n* = 625) were university graduates, while 18.4% (*n* = 435) were illiterate. History of COVID-19 infection was reported in only 7.7% (*n* = 182) of participants, while only 1.4% (*n* = 46) reported PCR-confirmed COVID-19 infections (Table 1).

**Table 1:** Socio-demographic data of 2360 participants according to results of their SARS-CoV-2 anti-S

	SARS-CoV-2 anti-S						*p*-value
	Negative		Positive		Borderline		
	No.	%	No.	%	No.	%	
Governorate							
Alexandria (30.3%)	396	55.4	295	41.3	24	3.4	0.236^$^
Monufia (27.0)	355	55.7	259	40.7	23	3.6	
Cairo (9.2%)	122	56.0	92	42.2	4	1.8	
Giza (5.5%)	64	49.2	64	49.2	2	1.5	
Qalyubia (8.8%)	123	59.4	77	37.2	7	3.4	
Dakahlia (10.9%)	150	58.1	92	35.7	16	6.2	
Faiyum (4.2%)	33	33.0	67	67.0	0	0.0	
Suez (4.0%)	21	22.1	74	77.9	0	0.0	
Gender							
Male (46.4%)	633	57.8	428	39.1	35	3.2	0.001*
Female (53.6%)	631	49.9	592	46.8	41	3.2	
Age (years)							
< 15 (19.4%)	241	52.7	208	45.5	8	1.8	0.328
15–29 (18.3%)	227	52.5	183	42.4	22	5.1	
30–39 (17.6%)	227	54.7	174	41.9	14	3.4	
40–59 (32.6%)	412	53.5	334	43.4	24	3.1	
60+ (12.1%)	157	54.9	121	42.3	8	2.8	
Residence							
Urban (51.2%)	630	52.1	552	45.7	27	2.2	0.008*
Rural (37.1%)	492	56.2	346	39.5	37	4.2	
Slum (11.7%)	142	51.4	122	44.2	12	4.3	
Education							
Illiterate (18.4%)	247	56.8	177	40.7	11	2.5	0.333
Primary (15.0%)	187	53.0	158	44.8	8	2.3	
Preparatory (12.7%)	150	50.2	140	46.8	9	3.0	
Secondary (27.5%)	331	51.1	291	44.9	26	4.0	
University (26.5%)	349	55.8	254	40.6	22	3.5	
Marital status							
Single (34.7%)	444	54.1	349	42.6	27	3.3	0.160^$^
Married (57.8%)	739	54.1	580	42.5	46	3.4	
Divorced/widow (7.4%)	81	46.3	91	52.0	3	1.7	
History of COVID-19 diagnosis^#^							
No (92.3%)	1223	56.2	885	40.6	70	3.2	0.001*
Yes (7.7%)	41	22.5	135	74.2	6	3.3	
History of PCR-confirmed COVID-19 infection							
No (98.6%)	1237	53.5	1001	43.3	76	3.3	0.410^$^
Yes (1.4%)	27	58.7	19	41.3	0	0.0	

^*^P < 0.05 (significant) using Pearson *X*^2^ test, except “$” which indicates the use of exact probability test

^#^Diagnosis based on any diagnostic means (clinical symptoms—laboratory biochemical and hematological parameters—rapid antigen test–PCR–chest CT)

**Fig. 1:**
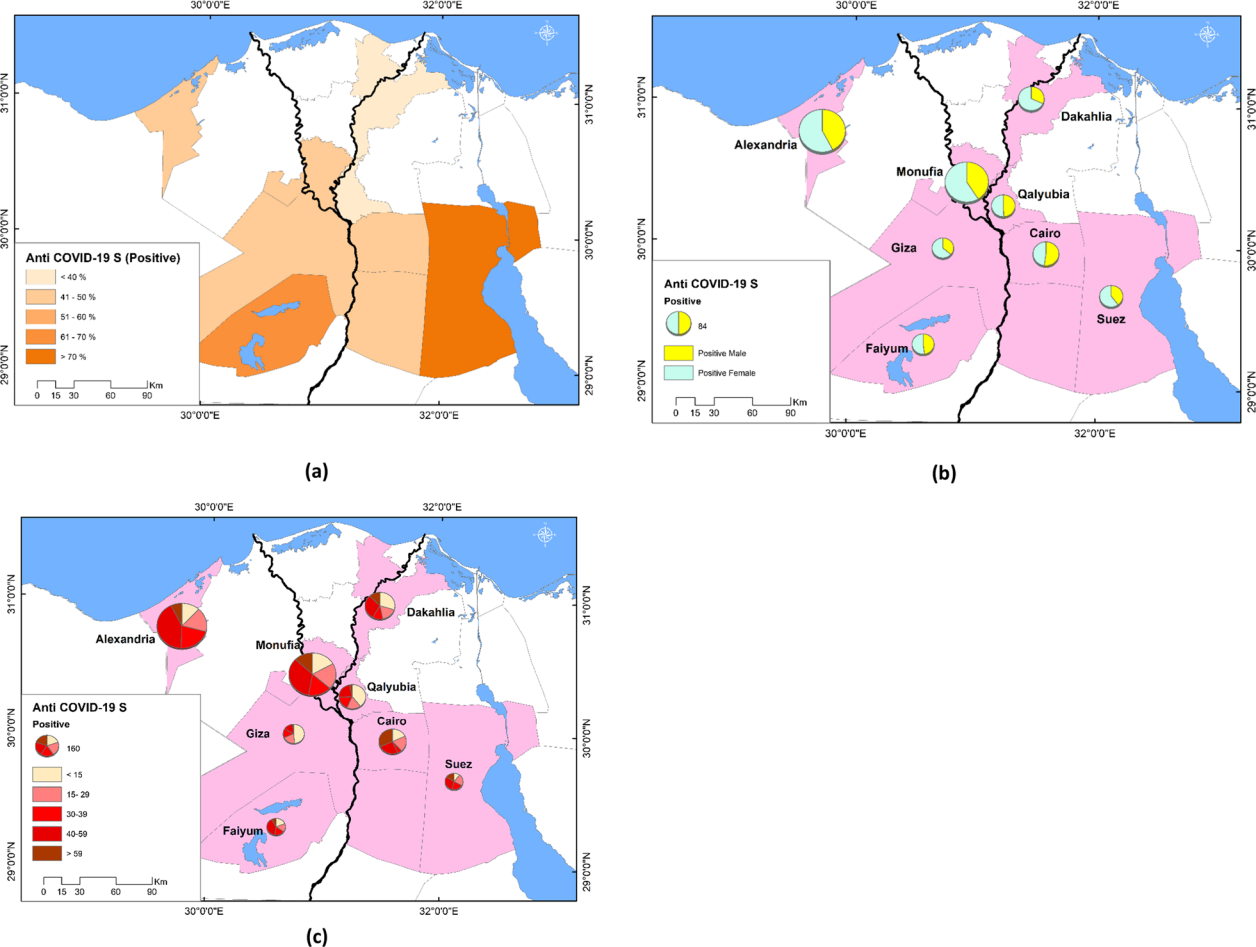
Seroprevalence of SARS-CoV-2 among 2360 participants in eight Egyptian governorates, 2021 **a** with gender, **b** and age, **c** distribution

The overall adjusted prevalence of anti-S among 2360 participants was 46.3% (95% CI 44.2–48.3%) Anti-S seroprevalence was highest in Suez, followed by Faiyum Governorate (77.9% and 67.0%, respectively), but differences between other governorates were insignificant. Gender, residence, and history of COVID-19 infection were significant determinants of anti-S positivity. Females had higher seropositivity compared to males (46.8% and 39.1%, respectively, *p* = 0.001). Regarding residence, 39.5% of rural residents were anti-S positive, while 45.7% of participants from urban areas were anti-S positive (*p* = 0.008). Positivity of anti-S was significantly higher among those reporting a history of COVID-19 infection compared to those who did not (74.2% and 40.6%, respectively, *p* = 0.001) (Table 1, Fig. 1a–c).

Cairo had the highest rate of PCR-confirmed COVID-19 cases (4.1%). In contrast, the rate of PCR utilization for diagnosis in the rest of the governorates was minimal compared to the utilization of laboratory tests or clinical-based diagnosis, ranging between 0–1% among the studied participants (*p* < 0.001) (Additional file 1: Table S1).

The overall adjusted prevalence of anti-S among 2360 participants was 46.3% (95% CI 44.2–48.3%) and was highest in Suez Governorate (83.6%; 95% CI 76.3–91.1%), followed by Faiyum (71.9%; 95% CI 62.9–80.8%), while it was lowest in Dakahlia Governorate (38.3%; 95% CI 28.4–48.2%). The estimated adjusted prevalence among males (41.9%; 95% CI 39–44.8%) was significantly lower than the prevalence among females (50.2%; 95% CI 47.4–53.0%). Considering the age of participants, the highest estimated prevalence for anti-S was among those aged < 15 years (48.8%; 95% CI 44.2–53.4%), with no significant difference between age groups. A history of COVID-19 infection was statistically associated with higher adjusted anti-S seroprevalence (79.6%; 95% CI 73.3–85.5%) (Table 2).

**Table 2:** Crude and adjusted prevalence of SARS-CoV-2 anti-S cases among 2360 participants from eight Egyptian governorates

	Crude prevalence		Adjusted prevalence	
	No.	%	Estimate%	95% CI
Governorate				
Alexandria	295	41.3	44.3	38.6–50.0
Monufia	259	40.7	43.6	37.6–49.5
Cairo	92	42.2	45.2	35.0–55.4
Giza	64	49.2	52.7	40.5–64.9
Qalyubia	77	37.2	39.8	28.9–50.7
Dakahlia	92	35.8	38.3	28.4–48.2
Faiyum	67	67.0	71.9	62.9–80.8
Suez	74	77.9	83.6	76.3–91.1
Gender				
Male	428	39.1	41.9	39.0–44.8
Female	592	46.8	50.2	47.4–53.0
Age (years)				
< 15	208	45.5	48.8	44.2–53.4
15–29	183	42.4	45.4	40.7–50.1
30–39	174	41.9	44.8	40.0–49.6
40–59	334	43.4	46.5	43.0–50.0
60+	121	42.3	45.2	39.4–50.9
Residence				
Urban	552	45.7	49.0	46.2–51.8
Rural	346	39.5	42.3	39.0–45.6
Slum	122	44.2	47.4	38.5–56.3
Educational level				
Illiterate	435	40.7	43.6	38.9–48.3
Primary	353	44.8	48.0	42.8–53.2
Preparatory	299	46.8	50.2	44.5–55.9
Secondary	648	44.9	48.1	44.3–51.9
University	625	40.6	43.5	39.6–47.4
Marital status				
Single	820	42.6	45.5	42.2–49.0
Married	1365	42.5	45.4	42.9–48.1
Divorced/widow	175	52.0	55.7	48.3–63.1
History of COVID-19 diagnosis^#^				
No	885	40.6	43.5	41.4–45.6
Yes	135	74.2	79.6	73.3–85.5
History of PCR-confirmed COVID-19				
No	1001	43.3	46.4	44.3–48.4
Yes	19	41.3	44.3	29.9–58.7
Overall				
Positive	1020	43.2	46.3	44.2–48.3

^#^Diagnosis based on any diagnostic means (clinical symptoms—laboratory biochemical and hematological parameters—rapid antigen test–PCR–chest CT)

The median anti-S titer among all seropositive participants was 39.0 RU/ml. Suez, followed by Faiyum Governorate, had the highest median anti-S titers (63.3 RU/ml and 56.3 RU/ml, respectively) while Dakahlia had the lowest (24.6 RU/ml). Age was a significant determinant of anti-S positivity (*p* < 0.001), where persons above 60 years of age had the highest median anti-S titer (66.9 RU/ml) followed by children younger than 15 years of age (53.15 RU/ml). Individuals with primary education had significantly higher anti-S titers compared to participants with other educational degrees (*p* = 0.009). Anti-S was also significantly higher among those reporting a history of COVID-19 infection (72.4 RU/ml versus 36.3 RU/ml, respectively, *p* < 0.001). Participants reporting previously diagnosed COVID-19 infection based on chest CT or PCR had significantly higher anti-S titers (120 RU/ml and 106.5 RU/ml, respectively, *p* < 0.001) compared to those diagnosed by other means of diagnosis (Additional file 1: Table S2).

## Discussion

Only 20–30% of COVID-19 patients are symptomatic, and only a smaller proportion of them undergo PCR testing. Due to the high expense of PCR testing, especially in low- and middle-income countries, the actual magnitude of COVID-19 prevalence in the community is largely indeterminate due to the limited numbers of tested samples [12]. COVID-19 surveillance in Egypt mainly depends on PCR tests, which are usually carried out in symptomatic cases presented at governmental hospitals [13, 14]. Results from private laboratories are not included in the official records of new cases and mortalities. This makes serological testing for antibodies a suitable surveillance tool for a more realistic estimation of viral spread in the community.

Cumulative COVID-19 infection and mortality rates escalate with time. Our study occurred during the 2nd and 3rd COVID-19 waves (January 2021 until the end of June 2021), and our results reflect the infection rates at that time. These figures have probably increased over time, with the wider spread of the pandemic in the country. In a meta-analysis, the overall average estimated pooled seroprevalence of anti-SARS-CoV-2 antibodies in Africa (between December 2020 and April 2021) was 22% (ranging from 0 to 63%), and this was close to the date of our present study (January 2021–June 2021) [15]. According to the WHO regional statistics, until the end of our study, the cumulative confirmed cases since the start of the pandemic in Egypt were 281,903 and mortalities reached 16,242 persons [16]. Using these official figures, the calculated rate for PCR-confirmed cases at that time would be 281,903/105 million population = 0.27%, the estimated overall mortality rate would be 16,242/105 million population = 0.015%, and the attributable mortality rate of COVID-19 would be 16,242/281,903 = 5.77%. However, our study reported that PCR-confirmed cases were 1.4% (which would be equivalent to 1,470,000 cases in the whole population), which is 5.2 folds higher than the official figures. This higher rate of PCR-confirmed cases is probably due to the inclusion in our study, of PCR-confirmed infections performed in private as well as governmental laboratories. On further analysis of these rates, when considering our PCR-confirmed cases to reflect the actual number of infections (regardless of the type of laboratory issuing the PCR result), the attributable mortality rate for COVID-19 might thus be lower than official rates (16,242/1,470,000 = 1.1%). According to an Egyptian study during the first two COVID-19 waves, the case fatality rate (CFR) declined from 9.22% in week 1 of the pandemic to 2.57% in weeks 9–10 [14]. In India, another study performed during the first two waves reported a CFR among PCR-confirmed cases of 2·4% [17]. These rates are higher than those estimated using our PCR results and might be affected by the performance of the healthcare system, population characteristics, circulating viral variants, and the burden of COVID-19 cases.

In our study, 7.7% of all participants reported a history of COVID-19 infection, including those persons diagnosed clinically, by laboratory investigations, radiologically as well as by PCR. This figure is a rough indicator of the burden of the disease in the community, given that not all patients had PCR tests done for them, owing to the scarcity of PCR tests at the time of the study. The infection-fatality rate would be substantially lower than the actual case fatality rate related to the lack of availability of PCR testing and inclusion of mild and asymptomatic infections. To the best of our knowledge, no similar data are available on the rates of COVID-19 infection in Egypt based on criteria for diagnosis (clinical symptoms/rapid test/laboratory tests) other than PCR.

According to a review article, the demographic details, clinical characteristics, and laboratory findings of Egyptian patients with COVID-19 showed variation between the first, second, and third waves regarding the incidence rate, the number of infected patients, and the hospitalization rates as well as some variations in patient characteristics [18]. In Egypt, Gomaa et al. followed up 1598 healthy participants for seven months (during the first wave of COVID-19 in Egypt) for the development of COVID-19, and the incidence of PCR-confirmed infection was 6.9% which is much higher than our rate (1.4%). This variation might be because Gomaa et al. included household cases and all their infected family members, thus increasing the rate of positive cases owing to household transmission. Moreover, they carried out their study in only four governorates (Gharbiyah, Kafr El-Sheikh, Qalyubiyah, and Faiyum) [19], while our study included eight governorates, which exceeds the number of governorates included by other authors.

The overall crude prevalence of anti-S among the 2360 participants was 43.2%. After adjustment for sensitivity and specificity, the seroprevalence for anti-S reached 46.3% (95% CI 44.2–48.3%). Gomaa et al. [19] reported that almost one-third of their participants from four Egyptian governorates were seropositive. However, their study was carried out during the first COVID-19 wave and detected neutralizing antibodies rather than anti-S, and thus their results should be expected to be lower than ours. Much lower seroprevalence rates were recorded in several studies elsewhere. In Switzerland, a sero-epidemiological study was conducted on 2766 households, and anti-S was positive in only 4.8–10.9% over the weeks of the study [20]. In France, anti-S seroprevalence (10%) was recorded in a large-scale study between May 4 and June 23, 2020 [21]. In China, a serological survey was conducted in seven cities between March 9 and April 10, 2020, on 10,499 individuals in the community, where seropositivity ranged from 0.6 to 3.8% among different cities, including Wuhan [22]. In comparison with other Arab countries, a national study on anti-S prevalence in several cities of Saudi Arabia showed a rate of 11%, with an apparent disparity between Saudi regions (Makkah had the highest rate at 24.4%) [23]. Generally, seroprevalence studies can be very different in terms of serological assays, sample source, geographical coverage, and population type; in addition, the timing of these studies may only reflect the dynamic transmission of the virus at the time [23]. Our high seroprevalence of anti-S IgG might imply the high sensitivity of our test kit and denotes high levels of viral infections (including asymptomatic and mild infections). Currently, it is still unclear which level of seropositivity correlates with viral elimination, however, increased seroprevalence denotes reduced risk of severe disease among the population, yet transmission is likely to continue because protection against infection appears to wane quickly. There is no known correlate of protection, a threshold above which people are protected from infection or severe disease. Higher titers are often found in convalescent patients and are thought to be protective against future infections [24], but also such high titers might be recorded during severe COVID-19 attacks [25]. Lower antibody titers are usually reported among asymptomatic cases and those with mild disease [9, 26]. According to the WHO, the proportion of the population that must be immune against COVID-19 to begin inducing herd immunity is not known [27]. An article published in *Nature* estimated that for herd immunity against COVID-19 to be attained, 60–70% of the population should be immune, either through vaccination or past exposure to the virus [28]. Our high seroprevalence in this study might be a step toward herd immunity but should be increased by higher vaccination rates. Until December 17, 2021, a total of 49,746,337 vaccine doses have been administered in Egypt [2]. Higher vaccination rates should be targeted, especially in governorates showing the least seroprevalence rates and lower anti-S levels, such as Dakahlia.

In our study, the adjusted prevalence of anti-S was highest in Suez Governorate (83.6%; 95% CI 76.3–91.1%), followed by Faiyum (adjusted prevalence: 71.9%; 95% CI 62.9–80.8%, respectively. The rest of the governorates all had similar and much lower seroprevalence. Cairo, the capital of Egypt, ranked fourth in anti-S rates (45.2%; 95% CI 35.0–55.4%), while Alexandria (the second-largest Egyptian governorate) ranked fifth. Cairo has the highest population per square kilometer (the most population-dense governorate) [29], yet several governorates exceeded Cairo’s seroprevalence rate, suggesting that other factors besides population density control the spread of SARS-Co-V-2 in the community. The least reported adjusted prevalence was in Dakahlia Governorate (38.3%; 95% CI 28.4–48.2%) and Qalyubia (39.8%; 95% CI 28.9–50.7%). These might be related to the differences between governorates regarding the socioeconomic and educational levels of residents, which might affect personal behaviors such as social distancing and wearing masks. The exceptionally high seroprevalence in Suez and Faiyum is striking and might be attributed to differences in exposure factors and adherence to precautionary measures. This manuscript does not explore such risk factors, but they are presented elsewhere [30]. Such variation in seroprevalence between governorates/cities was also reported in several countries, such as in Italy, which was among the most heavily affected countries, where the distribution of COVID-19 within the country varied extensively, with a notable gradient from the North to the South of Italy [31]. PCR utilization for diagnosis (at the time of the study) was highest in Cairo, followed by Alexandria, the two biggest cities in Egypt. More PCR utilization in smaller and remote governorates should thus be encouraged.

The median anti-S titer was calculated for seropositive cases only and was 39 RU/ml. Suez, followed by Faiyum Cairo Governorate, had the highest median anti-S titers (63.3 RU/ml, 56.3 RU/ml, and 51.4 RU/ml, respectively), while Dakahlia had the lowest (24.6 RU/ml). This pattern of anti-S titers is consistent with that of anti-S seropositivity and was of borderline statistical significance (*p* = 0.064). We suggest that governorates with low anti-S seroprevalence and titer levels, such as Dakahlia and Qalyubia, might increase their populations’ immune status through vaccination.

There was no significant difference between age groups regarding the prevalence of anti-S. In contrast, a study from Saudi Arabia reported lower anti-S seroprevalence in younger (below 18 years old) and older populations (older than 56 years) compared with other age groups (19–55 years) [32]. More frequent symptomatic or severe disease among elderly populations has also been hypothesized as a reason for higher anti-S titers [28]. In our study, although differences in seroprevalence between age groups were insignificant, anti-S titers were significantly higher among persons above 60 years of age (66.9 RU/ml) and children (< 15 years) (53.15 RU/ml) (*p* < 0.001), compared to middle-aged adults. Higher SARS-CoV-2 antibody titer among the elderly was reported by other studies [9, 33], which Wec et al. ascribed to the more frequent exposure to other human coronaviruses throughout the life of the elderly, which produces high levels of cross-reactive antibodies when patients are exposed to any of the human coronaviruses, including SARS-CoV-2 [33]. Such cross-reactivity with other coronaviruses occurs in all age groups but manifests most in the elderly due to their long time of exposure. Concerning the high titers of anti-S among children in our study, similar results were noted by Garrido et al., who reported higher anti-RBD and neutralizing antibodies in children than in adults, up to 4 months post-COVID-19 infection [34]. In addition to cross-reactivity with other coronaviruses, Karron et al., explained the higher SARS-CoV-2 seropositivity among children by their exaggerated tenfold higher RBD antibody titers than adults, while adults had a higher neutralizing ability than children [35]. Children were reported to have more durable, yet immature RBD-specific antibody responses [34].

“Being female” was a statistically significant determinant for anti-S positivity (adjusted prevalence of 50.2%; 95% CI 47.4–53.0% compared to 41.9%; 95% CI 39–44.8% among males). This suggests a more robust immune response by females. Wei et al. also reported that both genders were equally likely to seroconvert against the spike protein; however, among those who did, seroconvert males had a shorter IgG half-life than females [9]. In line with these findings, another study reported that females showed more robust T-cell activation and stronger antibody responses than males [36].

Living in an urban area was a significant factor for anti-S positivity (45.7% of urban residents were seropositive), and higher titers of anti-S (47.55 RU/ml) compared to those in rural or slum areas (*p* < 0.001). This might explain why Dakahlia Governorate (which had 99.6% of its participants living in rural areas) had the least anti-S adjusted prevalence (38.3%; 95% CI 28.4–48.2%) among all governorates. This finding might be due to the more population-dense nature of urban cities, with higher crowding and thus more infection rates. These findings are in accordance with those of another Egyptian study during the same period, where incidence rates were higher in urban compared to rural governorates (60.3 and 145.8/1,000,000 population, respectively) [14].

Educational level was also a significant determinant of seropositivity, where those with primary education had significantly higher anti-S titer (*p* = 0.009) levels compared to others with higher educational levels, denoting higher exposure and infection. A study on Saudi undergraduate students revealed that younger participants and people in their earlier academic years had low knowledge scores regarding COVID-19 modes of transmission and prevention, and the authors suggested that this might probably put them at a higher risk of contracting COVID-19 [37].

Seroconversion of anti-S spikes usually happens within 1–3 weeks after SARS-CoV-2 infection. However, 5–22% of individuals remain seronegative following infection [9]. In our study, among individuals reporting a history of COVID-19 diagnosis, anti-S adjusted seroprevalence was higher (79.6%; 95% CI 73.3–85.5%) and with higher titers (72.4 RU/ml) in comparison to 48.9% of others who did not report a history of infection (*p* = 0.001), and had significantly lower anti-S titers (36.3 RU/ml, respectively, *p* < 0.001). Regardless of the means of COVID-19 diagnosis, the seroprevalence of anti-S was significantly higher among persons reporting a previous infection.

On further analysis of the relationship between diagnostic method and antibody levels, diagnosis based on chest CT or PCR had significantly higher anti-S titers (120 RU/ml, and 106.5 RU/ml, respectively, *p* < 0.001) compared to other means of diagnosis (Additional file 1: Table S2), reflecting higher antibody production concerning viral load and lung affection. Similarly, Wei et al. reported higher seroconversion rates of anti-S to be associated with high viral loads [9].

## Limitations

Potential limitations of our study include the use of a convenience sample, which might have led more people with previous history of infection or those at high risk of infection to participate in our survey rather than those who were at low-risk and were not clinically diagnosed with COVID-19. If this was the case, our results might have overestimated the actual situation. Waning and potential seroconversion of infected individuals might have underestimated the actual burden of cases as well as the disease distribution of COVID-19 early on in the pandemic.

## Conclusions

Our study gives an overall view of the immune status of a representative population sample, reflecting their susceptibility to infection and associated host factors for seroprevalence. Exceptionally high seroprevalence of anti-S denotes high exposure to the virus as well as high immune response. Age, gender, residence, educational level, and previous PCR-confirmed COVID-19 infections were all determinants of the immune response. Governorates with lower overall anti-S rates might benefit most from higher COVID-19 vaccination coverage.

## Supplementary Information

**Additional file 1: Table S1.** Distribution of sociodemographic factors in 8 Egyptian governorates **Table S2.** Distribution of titer percentiles of 1020 participants positive for SARS-CoV-2.

## Data Availability

The datasets used and/or analyzed during the current study are available from the corresponding author on reasonable request.

## Abbreviations

COI: Cut-off index
CT: Computed tomography
ELISA: Enzyme-linked immunosorbent assay
IRB: Institutional Review Board
PCR: Polymerase chain reaction
RU: Relative unit
