# Correction: A pre-vaccine exploratory survey of SARS-CoV-2 humoral immunity among Egyptian general population

**DOI:** 10.1186/s41182-022-00457-w

**Published:** 2022-09-13

**Authors:** Engy Mohamed El-Ghitany, Shehata Farag, Azza Galal Farghaly, Mona H. Hashish, Mahmoud A. Hassaan, Eman A. Omran

**Affiliations:** 1 Department of Tropical Health and Parasitology, High Institute of Public Health, Alexandria University, 165 El-Horreya Avenue-El-Ibrahimia, Alexandria, Egypt; 2 Department of Biostatistics, High Institute of Public Health, Alexandria University, Alexandria, Egypt; 3 Family and Community Medicine Department, Faculty of Medicine, King Khalid University, Abha, Saudi Arabia; 4 Department of Microbiology, High Institute of Public Health, Alexandria University, Alexandria, Egypt; 5 Institute of Graduate Studies and Research, Alexandria University, Alexandria, Egypt

## Correction to: Tropical Medicine and Health (2022) 50:53 https://doi.org/10.1186/s41182-022-00448-x

Following publication of the original article [1], the authors reported an error in the fifth author affiliation, the city name should be Alexandria, Egypt instead of Abha, Egypt.

The correct author affiliation has been provided in this Correction.

The original article [1] has been corrected.
