# Supplementary material for: A pre-vaccine exploratory survey of SARS-CoV-2 humoral immunity among Egyptian general population
Source: Trop Med Health. 2022 Aug 10;50:53. doi: 10.1186/s41182-022-00448-x (PMC9364299; doi:10.1186/s41182-022-00448-x)
Supplement: Supplementary file 1 — Additional file 1: Table S1. Distribution of sociodemographic factors in 8 Egyptian governorates Table S2. Distribution of titer percentiles of 1020 participants positive for SARS-CoV-2. [file 41182_2022_448_MOESM1_ESM.docx]

**Table S1: Distribution of sociodemographic factors and means of COVID-19 diagnosis among 2360 participants from 8 Egyptian governorates, 2021**

| **Governorates** | | | | | | | | | | | | | | | | | | |
| --- | --- | --- | --- | --- | --- | --- | --- | --- | --- | --- | --- | --- | --- | --- | --- | --- | --- | --- |
|  | **Alexandria** | | | **Monufia** | | **Cairo** | | **Giza** | | **Qalyubia** | | **Dakahlia** | | **Faiyum** | | **Suez** | | P value |
|  | n | | % | n | % | n | % | n | % | n | % | n | % | n | % | n | % |  |
| **Gender** | Male | 306 | 42.8% | 317 | 49.8% | 125 | 57.3% | 59 | 45.4% | 107 | 51.7% | 93 | 35.8% | 49 | 49.0% | 40 | 42.1% | <0.001 |
|  | Female | 409 | 57.2% | 320 | 50.2% | 93 | 42.7% | 71 | 54.6% | 100 | 48.3% | 165 | 64.2% | 51 | 51.0% | 55 | 57.9% |  |
| **Age (years)** | < 15 | 108 | 15.1% | 102 | 16.0% | 34 | 15.6% | 56 | 43.1% | 57 | 27.5% | 72 | 28.0% | 18 | 18.0% | 10 | 10.5% | <0.001 |
|  | 15-29 | 115 | 16.1% | 121 | 19.0% | 41 | 18.8% | 29 | 22.3% | 51 | 24.6% | 44 | 17.1% | 15 | 15.0% | 16 | 16.8% |  |
|  | 30-39 | 153 | 21.4% | 108 | 17.0% | 19 | 8.7% | 18 | 13.8% | 38 | 18.4% | 35 | 13.6% | 20 | 20.0% | 24 | 25.3% |  |
|  | 40-59 | 282 | 39.4% | 222 | 34.9% | 61 | 28.0% | 19 | 14.6% | 49 | 23.7% | 73 | 28.0% | 37 | 37.0% | 27 | 28.4% |  |
|  | 60+ | 57 | 8.0% | 84 | 13.2% | 63 | 28.9% | 8 | 6.2% | 12 | 5.8% | 34 | 13.2% | 10 | 10.0% | 18 | 18.9% |  |
| **Residence** | Urban | 438 | 61.3% | 176 | 27.6% | 213 | 97.7% | 2 | 1.5% | 192 | 92.8% | 2 | 0.4% | 91 | 91.0% | 95 | 100.0% | <0.001*^$^* |
|  | Rural | 7 | 1.0% | 461 | 72.4% | 3 | 1.4% | 127 | 97.7% | 12 | 5.8% | 256 | 99.6% | 9 | 9.0% | 0 | 0.0% |  |
|  | Slum | 270 | 37.8% | 0 | 0.0% | 2 | 0.9% | 1 | 0.8% | 3 | 1.4% | 0 | 0.0% | 0 | 0.0% | 0 | 0.0% |  |
| **Marital status** | Single | 238 | 33.3% | 185 | 29.0% | 70 | 32.1% | 80 | 61.5% | 96 | 46.4% | 97 | 37.7% | 29 | 29.0% | 25 | 26.3% | <0.001*^$^* |
|  | Married | 423 | 59.2% | 408 | 64.1% | 119 | 54.6% | 44 | 33.8% | 101 | 48.8% | 138 | 53.3% | 66 | 66.0% | 66 | 69.5% |  |
|  | Divorced / widow | 54 | 7.6% | 44 | 6.9% | 29 | 13.3% | 6 | 4.6% | 10 | 4.8% | 23 | 8.9% | 5 | 5.0% | 4 | 4.2% |  |
| **Education** | Illiterate | 97 | 13.6% | 117 | 18.4% | 30 | 13.8% | 38 | 29.2% | 22 | 10.6% | 81 | 31.5% | 31 | 31.0% | 19 | 20.0% | <0.001 |
|  | Primary | 95 | 13.3% | 83 | 13.0% | 26 | 11.9% | 34 | 26.2% | 46 | 22.2% | 47 | 18.3% | 11 | 11.0% | 11 | 11.6% |  |
|  | Preparatory | 77 | 10.8% | 74 | 11.6% | 21 | 9.6% | 33 | 25.4% | 32 | 15.5% | 28 | 10.9% | 17 | 17.0% | 17 | 17.9% |  |
|  | Secondary | 138 | 19.3% | 224 | 35.2% | 54 | 24.8% | 17 | 13.1% | 65 | 31.4% | 86 | 33.5% | 31 | 31.0% | 33 | 34.7% |  |
|  | University | 308 | 43.1% | 139 | 21.8% | 87 | 39.9% | 8 | 6.2% | 42 | 20.3% | 16 | 5.8% | 10 | 10.0% | 15 | 15.8% |  |
| **Means of COVID-19 diagnosis** | Never | 640 | 89.5% | 584 | 91.7% | 193 | 88.5% | 127 | 97.7% | 196 | 94.7% | 257 | 99.6% | 94 | 94% | 87 | 91.6% | <0.001*^$^* |
|  | PCR | 18 | 2.5% | 3 | 0.5% | 9 | 4.1% | 1 | 0.8% | 1 | 0.5% | 0 | 0.0% | 1 | 1% | 1 | 1.1% |  |
|  | Rapid antigen test | 2 | 0.3% | 8 | 1.3% | 0 | 0.0% | 0 | 0.0% | 0 | 0.0% | 0 | 0.0% | 0 | 0.0% | 0 | 0.0% |  |
|  | lab tests | 30 | 4.2% | 35 | 5.5% | 8 | 3.7% | 0 | 0.0% | 3 | 1.4% | 0 | 0.0% | 3 | 3.0% | 4 | 4.2% |  |
|  | chest CT | 7 | 1.0% | 0 | 0.0% | 1 | 0.5% | 0 | 0.0% | 3 | 1.4% | 0 | 0.0% | 0 | 0.0% | 2 | 2.1% |  |
|  | Clinical symptoms | 18 | 2.5% | 7 | 1.1% | 7 | 3.2% | 2 | 1.5% | 4 | 1.9% | 1 | 0.4% | 2 | 2.0% | 1 | 1.1% |  |

** P < 0.05 (significant) using Pearson X^2^ test, except “$” which indicates the use of Exact probability test*

**Table S2: Distribution of titer percentiles of 1020 participants positive for SARS-CoV-2 anti-S from 8 Egyptian Governorates**

| **Risk factors** | | **Anti-S titer (RU/ml )** | | | **p-value** |
| --- | --- | --- | --- | --- | --- |
|  |  | **25^th^ percentile** | **50^th^ percentile** | **75^th^ percentile** |  |
| **Governorate** | Alexandria | 22.50 | 40.40 | 88.70 | 0.064 |
|  | [Monufia](https://en.wikipedia.org/wiki/Monufia_Governorate) | 21.40 | 36.30 | 74.30 |  |
|  | Cairo | 27.05 | 51.40 | 94.40 |  |
|  | Giza | 19.15 | 42.45 | 73.10 |  |
|  | [Qalyubia](https://en.wikipedia.org/wiki/Qalyubia_Governorate) | 19.60 | 40.40 | 72.70 |  |
|  | [Dakahlia](https://en.wikipedia.org/wiki/Dakahlia_Governorate) | 16.60 | 24.60 | 55.30 |  |
|  | Faiyum | 30.20 | 56.30 | 113.75 |  |
|  | Suez | 35.60 | 63.30 | 115.20 |  |
| **Gender** | Male | 19.30 | 38.00 | 73.70 | 0.513 |
|  | Female | 21.82 | 39.90 | 76.17 |  |
| **Age ( years)** | < 15 | 28.27 | 53.15 | 77.50 | <0.001* |
|  | 15-29 | 18.50 | 28.40 | 53.90 |  |
|  | 30-39 | 17.70 | 29.70 | 63.00 |  |
|  | 40-59 | 21.60 | 37.80 | 75.00 |  |
|  | 60+ | 25.07 | 66.90 | 120.00 |  |
| **Residence** | Urban | 22.82 | 47.55 | 88.92 | <0.001* |
|  | Rural | 18.00 | 33.10 | 65.40 |  |
|  | Slum | 23.35 | 36.05 | 68.52 |  |
| **Educational level** | Illiterate | 20.85 | 37.50 | 68.95 | 0.009* |
|  | Primary | 28.55 | 52.75 | 85.65 |  |
|  | Preparatory | 22.00 | 37.75 | 64.30 |  |
|  | Secondary | 18.80 | 33.50 | 74.90 |  |
|  | University | 19.20 | 39.80 | 81.10 |  |
| **Marital status** | Single | 21.50 | 42.20 | 74.30 | 0.008* |
|  | Married | 19.50 | 35.70 | 74.70 |  |
|  | Divorced / widow | 27.50 | 51.80 | 98.80 |  |
| **History of COVID-19 diagnosis** | No | 19.80 | 36.30 | 68.67 | <0.001* |
|  | Yes | 33.50 | 72.40 | 120.00 |  |
| **Means of COVID-19 diagnosis (n=118)** | Clinical symptoms | 27.10 | 46.70 | 67.80 | <0.001* |
|  | Biochemical/hematological laboratory tests | 31.85 | 73.90 | 120.00 |  |
|  | Rapid Antigen test | 25.20 | 81.20 | 120.00 |  |
|  | PCR | 39.80 | 106.50 | 120.00 |  |
|  | Chest CT | 84.60 | 120.00 | 120.00 |  |
| **Total** | | 20.90 | 39.00 | 74.80 |  |

** P < 0.05 (significant) using Kruskal Wallis test*
